# Current Trends in Liquid Biopsy Tracking Resistance in Molecular Breast Cancer-Targeted Therapies

**DOI:** 10.3390/genes16040443

**Published:** 2025-04-09

**Authors:** Richard Tancredi, Navid Sobhani, Martina Catalano, Giandomenico Roviello, Daniele Generali

**Affiliations:** 1 Multidisciplinary Unit of Breast Pathology and Translational Research, Cremona Hospital, 26100 Cremona, Italy; rictanc@hotmail.com; 2 Department of Cancer Biology, The University of Texas MD Anderson Cancer Center, Houston, TX 77030, USA; navid.sobhani@cantab.net; 3 Department of Health Sciences, Section of Clinical Pharmacology and Oncology, University of Florence, 50139 Florence, Italy; martina.catalano@unifi.it; 4 Department of Medicine, Surgery and Health Sciences, University of Trieste, 34100 Trieste, Italy; dgenerali@units.it

**Keywords:** liquid biopsy, ctDNA, CTC, biomarkers, breast cancer

## Abstract

Breast cancer (BC) is the most commonly occurring type of cancer in women, being a major cancer-related cause of mortality worldwide. With the advancement in current therapeutic options, including hormone therapy and targeted therapies, there is a need for more accurate and less invasive options to monitor cancer progression in patients. Liquid biopsy has evolved rapidly, being able to detect small quantities of nucleic acids or cell-free DNA in the blood of BC patients. This method addresses three major issues of needle biopsy: firstly, it is more permissive by being less invasive and does not require needling the organs; secondly, it covers for the heterogeneous nature of the tumor of origin, which could lead to an otherwise inaccurate representation of the cancer-driving mutations; thirdly, it better represents the type of tumor that the primary tumor is going to evolve into before it starts to metastasize. This current review will address the current advancements in liquid biopsy in the context of BC, highlighting the pros and challenges.

## 1. Introduction

Almost 12% of the cancers that occur in women worldwide are breast cancer (BC), making it second only to lung cancer in incidence [1]. In 2022, 665,684 women died of BC, which ranks fourth in cancer mortality around the globe [2]. Based on different receptor expressions, BC can be divided into four different subtypes: two luminal subtypes (luminal A and luminal B), the human epidermal growth factor receptor 2 (HER2) subtype, and triple-negative BC (TNBC), which does not express any of these three receptors [3]. Overall, ER-positive and progesterone receptor (PR)-positive breast cancer are called hormone receptor-positive (HR-positive) cancers, which make up 70% of all breast cancer malignancies [3].

Briefly, in hormone receptor-positive (HR+) BC, endocrine therapy (ET) is the base of the first-line therapy. HER2+ patients are treated by adjuvant antibody trastuzumab-based therapy, targeting the HER2 receptor [4]. Antibodies conjugated with chemotherapies targeting HER2+ BC cells have been successfully tested. However, in TNBC where there are no receptors to target, alternative therapies are most needed [5]. The discovery of alterations in the levels of the CDK4/6 pathway in the context of breast cell proliferation and cancer [6,7] led to the discovery of new CDK4/6 inhibitors that have been dramatically modified for better BC therapy and the possibility for TNBC [8]. Additionally, CDK4/6 inhibitors are usually used together with ET in HR+ BC. This has become the standard of treatment. Palbociclib, ribociclib, and abemaciclib are three FDA-approved CDK4/6 inhibitors that are approved to be used together with ET in HR+ Her2-negative BC. Their chemistries and functions are similar [9]. These cyclin-dependent kinases play essential roles in controlling proper cell cycle progression and proliferation [6], especially when the cell has to transition from the G1 phase into the S phase. Unfortunately, current statistics show that one out of ten patients eventually become resistant to these very efficient inhibitors. As a consequence, in this subset of patients, the drug will stop working [10]. There is a need for diagnostic methods that can predict in time if patients will relapse or become resistant to therapy.

Liquid biopsy can be used for this purpose. It can be used to find the level of any gene. It can also detect immunotherapy biomarkers, such as PD-L1. This comes particularly useful for the very aggressive type, TNBC. MicroRNAs that can inhibit TNBCs have been developed [11]. Moreover, PD-L1 has been detected on CTCs, exosomes, plasma, and PBMCs. There are currently six clinical studies that have investigated PD-L1 expression from CTCs, EVs, and soluble samples of BC patients treated with immunotherapy [12,13,14,15,16,17]. For a review of PD-L1 in liquid biopsy of BC, please refer to this review: [18].

**Figure 1:**
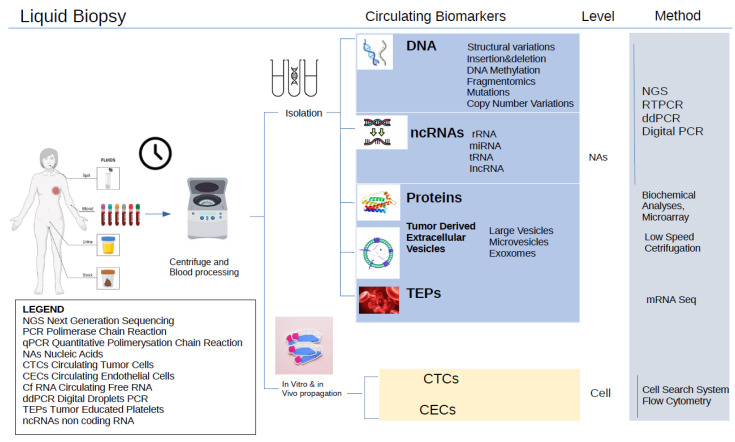
Liquid biopsy for breast cancer. It requires minimally invasive blood extraction.

Mechanisms of resistance to BC therapies include drug degradation by enzymes and changes in hormone receptors’ absorption, transportation, and efflux. In HER2+ BC patients, the expression of the HER2 receptor could lower over time, making the drugs against it inefficient against cancer [19]. Similarly, a reduction in ER+ could be detrimental to hormone therapies. Therefore, having a method that can quickly detect such changes is crucial. (Figure 1) [20,21,22,23].

There is still an unmet need in breast cancer management, which is to be able to frequently monitor treatment efficacy without using invasive needle methods or always resorting to X-rays. Liquid biopsy has been at the forefront in this effort. Since tumors are heterogeneous in nature, using needle biopsy methods can only be representative of the small region of cancer being extracted, and sometimes, even that region could have more normal or fat tissues, compromising the assessment. The advantage of using liquid biopsy is that the composition of the tumor shed into the blood is full detected. We examined the literature for the use of liquid biopsy in breast cancer (Supplementary Figure S1).

Liquid biopsy in breast cancer has evolved considerably since its inception in the year 2006 [24,25]. From a few tests, kits have been gradually developed, with the first one being FDA-approved for solid tumors, namely, the FoundationOne Liquid CDx, which analyses 324 genes from a simple blood draw [26]. Guardant360 CDx is another FDA-approved CpG panel for targeted BC-targeted therapy with ESR1 mutations [27]. The development of more kits will help guide clinicians to give the best therapies and predict recurrence. Liquid biopsies look at blood, cerebrospinal fluid, and urine for tumor molecules. The most common liquid biopsies look at circulating tumor DNA/microRNA (ctDNA/ctmiRNA) and circulating tumor cells (CTCs). CTCs circulate through the bloodstream to find other locations for metastasis, or they are passively shed from either the original tumor or the tumor at the metastatic location [28]. Among the various methods of detecting tumors in circulation is cell-free DNA (cfDNA), which is a key component of liquid biopsy. There are other types of genetic tumor material besides DNA. Although cell-free messenger RNA is very unstable, there are also ways to detect it. Additionally, miRNAs, a type of noncoding RNA that can regulate the expression of genes, are more common molecules that can be detected in the blood [29]. Exosomes could contain miRNA, mRNA, and DNA of tumor origin. These vesicles have the advantage of protecting their cargo because they have a membrane. They can be shed from the membranes, usually through a process of exocytosis, in which they fuse with the membrane before being released.

**Figure 2:**
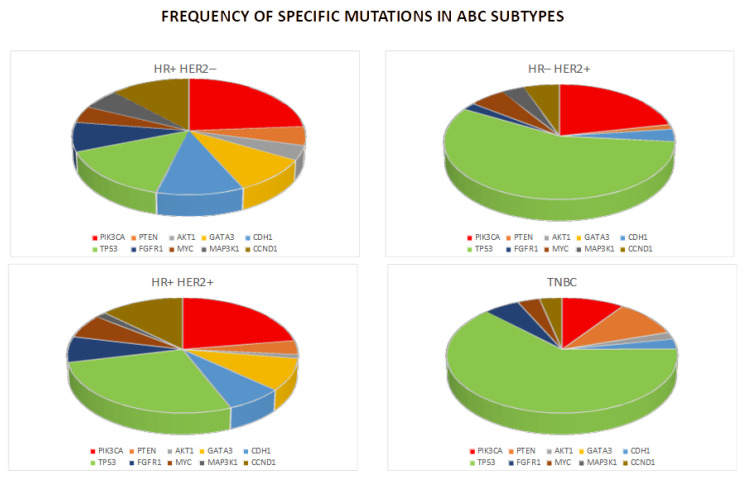
Chart of the frequencies of the mutations in breast cancer subtypes.

Compared to needle biopsies, liquid biopsies have several advantages: (1) they are less invasive; (2) they can sample the tumor more comprehensively, thus taking into account the tumor heterogenicity; (3) they can capture the genetic texture of multiple tumors at the same time; and (4) they can give a real-time assessment of the evolution of the tumor biology and the patient’s response to treatment [23] based on different mutations (Figure 2). In this review, we examine the use of liquid biopsy in breast cancer for early diagnosis, detection of minimal residual disease, prediction of recurrence, and assessment of treatment response to help best guide clinical decisions.

### 1.1. Early Diagnosis

Very encouraging results have been observed in studies of non-invasive early detection of CTCs in breast cancer. The implication of this detection is the capacity to envision the possible presence of primary tumors. Kruspe et al. [29] showed that probed nuclease-derived CTCs could distinguish patients who would develop metastases from healthy controls. Data from the PREDICT trial showed that CTCs were able to predict OS independently from treatment and tumor subtype [29,30]. A caveat to this method is that CTCs are present at low levels when the disease is still at an early stage, making it challenging to detect them and, thus, detect the tumor at its very early moments or before it even develops.

### 1.1.1. cfDNA

cfDNA is an early detection biomarker that is being compared in some breast cancer studies with changes in DNA damage as well as changes in DNA methylation status. Early-stage tumors release less ctDNA compared to advanced cancers, making their detection challenging, but potentially crucial, for early diagnosis. Identifying specific mutations, methylation patterns, or other alterations in the ctDNA genes using liquid biopsy can detect the presence of cancerous changes before they are visible on imaging.

Agostini et al. [31] developed a cfDNA integrity index based on the observation that tumor cells release larger fragments of cfDNA because of tumor necrosis compared with the smaller pieces of cfDNA released by normal cells during apoptosis. The baseline levels of the DNA repetitive element ALU247 (cfDNA tumoral fragments of 247 base pairs through the ALU247 PCR primers) were shown to be significantly higher in cancer patients and were precise and accurate in discriminating between patients and non-cancer controls. ALU247 levels were significantly higher in 11 patients with metastatic lymph nodes than in 22 healthy controls. Another DNA repetitive element is LINE-1 (cfDNA tumor fragments of 247 base pairs with the employment of ALU247 PCR primers). These repetitive elements could be used to calculate cfDNA integrity. For example, using the ratios of ALU 260/111 and of LINE-1 266/97, significantly lower cfDNA integrity was detected in recurrent breast cancer compared to non-recurrent breast cancer (n = 37 vs. n = 175, respectively, *p* < 0.001 for both ALU 260/111 and LINE-1 266/97 cfDNA integrity values) [23,32].

Apart from ALU247 and LINE-1, other cfDNA markers are four polymorphic markers showing loss of heterogenicity (LOH): D13S159, D13S280, and D13S282 at region 13q31-33 and D10S1765 at *PTEN* region 10q23.31. These markers can be measured using polymerase chain reaction (PCR)-based fluorescence microsatellite analyses. Interestingly, they were also significantly correlated with the status of the lymph nodes [33]. Besides cfDNA quantity, integrity, and LOH, in cfDNA there is a statistical difference in the single-nucleotide polymorphisms (SNPs) in the normal leukocytes of patients with breast cancer compared with those of normal controls. These SNPs could, therefore, also play an important role in breast cancer prognosis [33].

### 1.1.2. ctDNA

In contrast to cfDNA, ctDNA has been observed in smaller fractions and is restricted to localized tumors. Amplification-refractory *PI3KCA* mutations were detected by Board et al. [34] through system allele-specific and Scorpion PCR probes in the ctDNA of 80% of metastatic breast cancer patients having *PIK3CA* mutations. Similarly, ctDNA was detected by Bettegowda et al. [35] in half of patients with localized breast cancer using technologies that are highly sensitive. Such methods included PCR ligation, BEAMing (beads, emulsification, amplification, and magnetics), and massively parallel sequencing. Unfortunately, the prediction values varied across these studies, reflecting the differences in sensitivity across ctDNA assays. This evinces the criticality of establishing consensus on a standardized ctDNA detection method that is powerful.

Specific mutations in breast cancer, including breast cancer-specific biomarkers, can be detected from ctDNA. CancerSEEK is a pan-cancer blood test specifically designed to recognize biomarkers of eight different cancer types, including breast cancer. This method has a panel of 16 different ctDNA genes, including 7 of the most common ones: *TP53*, *PIK3CA*, *APC*, *PTEN*, *KPAS*, *NRAS*, and *CTNNB1* [36,37].

Oncogene PIK3CA is found to be highly mutated in almost one-third of breast cancer cases. PIK3CA mutations were evaluated by Beaver et al. [38] in the plasma of patients with breast cancer. The authors observed that the sensitivity and specificity of PIK3CA for detecting early-stage breast cancer were 93.3% and 100%, respectively [37]. There are limitations in the use of ctDNA in early-stage tumor detection, related to low signal-to-noise ratios. This could be readily addressed by implementing more powerful tools, like using the latest digital PCR methods offered by leading companies in the field, capable of detecting from 1 to 10 ng of DNA material per patient’s blood sample, to detect ctDNA or multiplex up to ten genes at a time. This research could also prove insightful in solving the low ratio issue, while testing additional comprehensive biomarker approaches like tumor-derived exosomes or methylated RNA. Recently, in 119 TNBC patients, a tissue-free epigenomic ctDNA-based assay was able to detect poor prognosis during neoadjuvant setting [38].

Another way of detecting early breast cancer comes from miRNA. miRNA expression profiles were evaluated by Erbes et al. [39] in patients with breast cancer vs. healthy controls. The authors identified five miRNAs (miR-1246, miR-1307-3p, miR-4634, miR-6861-5p, and miR-6875-5p) whose expression enabled the detection of breast cancer with high sensitivity (97.3%), specificity (82.9%), and accuracy (89.7%). Carcinoma in situ was detected in individuals using this method, showing 98.0% sensitivity. An initial study looking at both blood and urine samples of patients with breast cancer [36] identified differential profiles of four circulating miRNA: miR-21, miR-451, miR-125b, and miR-155. These markers could discriminate between women with breast cancer and healthy women. The analysis of urine samples in this study was found to be reliable, robust, and reproducible. The expression profile of four other ctmiRNAs (miR-423, miR-424, miR-660, and let7-i) was detected by Hirschfeld et al. [40] in the urine of breast cancer patients. Once again, this non-invasive method showed promise by being able to properly differentiate between patients with breast cancer and healthy controls.

### 1.1.3. RNA

Plasma exosome-derived long-noncoding RNAs (lncRNAs), which are abundant in many cancer types including breast cancer, are also a promising biomarker [28,29]. Zhong et al. [41] analyzed lncRNA H19 derived from exosomes in sera. H19 is an oncogene that has been found to drive cell growth, tumor invasion, and apoptotic pathways. It has been previously reported as a biomarker capable of predicting breast cancer progression [42]. The authors found that the expression of exosomal H19 was significantly higher in breast cancer patients compared to healthy controls.

Another blood biomarker for breast cancer diagnosis is tumor-educated platelets (TEPs). In a study of six different tumor types, including breast cancer, conducted by Best et al. [43], TEPs were able to identify the locations of primary tumors with an accuracy of 71%. Different stimuli are induced by different molecular subtypes of breast cancer, and the TEP profiles were also able to successfully distinguish between patients with amplification of *PI3KCA*, *HER2* mutation, and triple-negative breast cancer phenotypes. TEP cfRNA and RNA are also being widely studied, as their simplicity in detection makes them potentially useful biomarkers. The in-depth analysis of circulating RNA has evolved considerably, from microarray sequencing techniques to in-depth sequencing to confirmation with qRT-PCR.

mRNA and proteins have also been studied as biomarkers that can be identified in liquid biopsy. Eight mRNAs (*S100A8*, *TPT1*, *GRIK1*, *CSTA*, *GRM1*, *IGF2BP1*, *H6PD*, and *MDM4*) and carbonic anhydrase VI (CA6) were investigated by Zhang et al. [44]. Both the eight mRNAs and the CA6 protein were able to distinguish successfully between breast cancer patients and healthy controls. The authors reported 92% accuracy, 83% sensitivity, and 97% specificity.

### 1.1.4. DNA Methylation

Another well-characterized epigenetic method for gene regulation is DNA methylation. The genome is globally hypomethylated but has focal hypermethylations of 5′-cytosine–phosphate–guanine-3′ (CpG) islands in large numbers. Such islands span across gene promoters and the first exons. Aberrant methylation in both tissue and liquid biopsies is correlated with carcinogenesis and cancer progression [33]. The early prediction of breast cancer could be improved significantly by using a combination of multiple biomarkers, such as ctDNA, miRNA, exosome-derived RNA, RNA methylation panels, and TEPs, instead of the single biomarkers. These panels should be tested in both observation and prospective clinical trials. Moreover, very sensitive technologies, such as dPCR and NGS could detect low ctDNA at early stages of cancer development.

In summary, various studies have suggested that having early diagnosis biomarkers, from the blood, urine, or even breath of patients, would corroborate mammogram methods determining possible tumors in the breast or, better still, be able to predict tumor formation, therefore helping to prepare clinicians for the right therapy in advance.

### 1.2. Minimal Residual Disease and Predicting Recurrence

Minimal residual disease (MRD) refers to the small number of tumor cells that remain in the body after treatment. Liquid biopsy has been proven to effectively characterize and detect MRD. It can identify tumor cells moving from the original primary tumor location to distant organs, even in patients who do not show clear radiological signs of metastasis. It can also detect residual tumor cells that have abandoned their initial location after local therapy, which can eventually lead to local recurrence. In the nonmetastatic setting, the monitoring of ctDNA after surgery and neoadjuvant treatment can help assess MRD and relapse risk.

In a retrospective study of breast cancer with long-term follow-up, two approaches were used to detect genetic material from the liquid biopsy. The first approach was whole-genome sequencing with low coverage for the primary tumor. The second approach was quantification through droplet digital PCR of tumor-specific genetic rearrangements in the patient’s plasma. Using these approaches, post-surgery ctDNA monitoring could accurately discriminate between patients with metastasis and those without it with 93% sensitivity, 100% specificity, and an operator curve area of 0.98 (*p* < 0.001). Additionally, ctDNA was able to be detected before clinical doctors could identify the formation of metastasis in 86% of patients. With 11 months (range, 0 to 37 months) of average follow up, patients without metastasis or disease recurrence did not have detectable ctDNA postoperatively.

*RASSF1* gene methylation was studied in ctDNA in another study, and it was shown to be significantly lower in patients with breast cancer who responded to neoadjuvant therapy than in those who did not (*p* < 0.006) [45]. None of the 47 patients with ctDNA-negative methylation status showed tumor recurrence over a follow-up time of 23 months (range, 3 to 33 months). The absence of the methylation of *RASSF1* after neoadjuvant therapy predicted pathological complete response with higher accuracy than ultrasonography or mammography did [46]. On the other hand, the detection of ctDNA in the plasma after neoadjuvant chemotherapy and surgery could predict the recurrence of metastasis with a median time of around 7.9 months [47]. Being able to sequence ctDNA instead of the primary tumor could lead to a more accurate molecular profile of the cancer and prediction of subsequent relapse. Such data provide a clearer understanding of how to treat relapsed cancer [48]. There is no consensus on the correlation between ctDNA values in BC patients prognosis and response to therapy. The majority of clinical trials have considered higher ctDNA levels correlated with worse prognosis and therapy response. These discrepancies could be due to a variety of factors ctDNA detection (e.g., qPCR, dPCR, or electrophoretic separation), different lengths of ALU and LINE1 fragments being considered for PCR, the prospective or retrospective nature of the study, the different populations’ ages, and the type of therapy. Additionally, another challenge could be that tumor shedding into the blood circulation may occur slowly, and during certain intervals, it might occur even at quantities below what is considered minimal. Consequently, the tumor-derived DNA could be temporarily undetected in the blood circulation with standard methods of detection, thereby delaying crucial, timely MRD detection. Briefly, the relevance of liquid biopsy remains due to the fact that solid tumors are very heterogenous. Needle biopsy might sample a part of the tumor mass that is not completely representative of the tumor’s molecular composition. On the other hand, liquid detects, more broadly, the presence of tumor cells in the blood of the patient, thereby predicting recurrence.

### 1.3. Prediction of Treatment Response to Specific Therapies

Liquid biopsy can be used to predict treatment responses in breast cancer. The next section will mainly focus on the anti-*ESR1* therapy as the best example for explaining this application of liquid biopsy [49,50,51]. Early-stage breast cancer patients whose tumors had been shed from the tumor of origin into the blood circulation after receiving adjuvant aromatase inhibitor (AI) therapy were studied by Allouchery et al. [52]. *ESR1* mutation in ctDNA could not be detected after adjuvant AI therapy. However, after patients experienced their first relapse, 5.3% (2/38) of them had mutated *ESR1* in ctDNA, and when the metastatic tumor was treated for the first time, 33% (7/21) of the patients receiving AI had mutated *ESR1* in ctDNA, compared to zero patients who received chemotherapy. As an overall observation, mutated *ESR1* in ctDNA seems to be more prevalent in patients receiving AI during metastasis than in those receiving adjuvant treatment, which indicates that this marker could be used to gradually assess tumor progression in subsequent therapeutic settings.

The difference in the presence of *ESR1* mutation in ctDNA across different patients receiving either adjuvant AI or AI at metastasis was shown to be statistically significant (5.8%, 3/52 patients vs. 36.4%, 16/44 patients, *p* < 0.001) by Schiavon et al. [53]. It is noteworthy that patients who did not receive AI in this study did not have detectable *ESR1* mutations in their ctDNA.

Shorter PFS was also observed in patients with metastatic breast cancer who had ctDNA *ESR1* mutations after receiving AI [53]. The hazard ratio was 3.1, with a 95% confidence interval of 1.9–23.1 and *p* < 0.004. Therefore, this study showed that there is a link between resistance to AI therapy and circulating *ESR1* mutations.

The concordance between the ctDNA *ESR1* status and metastatic tissue was calculated to be 47%, whereas the concordance between ctDNA *ESR1* status and primary tissue was only 5%, in a study by Spoerke et al. [54]. After and before AI administration, the concordances were 57% and 23%, respectively. Before AI therapy, *ESR1* mutations were found in 3.7% (3/81) of patients, compared with 57% (12/21) of patients after progression to AI therapy. *ESR1*-mutation-bearing patients had significantly worse survival outcomes after receiving AI therapy. Through ddPCR, it was found that the allele frequency of the mutated *ESR1* ctDNA correlated with endocrine therapy resistance and disease progression in three of four patients in a study conducted by Wang et al. [55].

In a meta-analysis of CTC studies in patients with ER-positive metastatic breast cancer receiving endocrine therapy, *ESR1* mutational status was compared between CTCs and ctDNA [56]. Two cohorts were generated: one cohort had 43 patients receiving first-line endocrine therapy and a second cohort had 40 patients who had progressive disease after first or later lines of endocrine therapy. The key finding was that *ESR1* ctDNA mutations showed higher prevalence in the disease progression cohort compared to the baseline one (42% vs. 11%; *p* < 0.04). This observation suggests that *ESR1* plays a strong role in resistance to endocrine therapy in metastatic breast cancer. Interestingly, the *ESR1* mutations behaved differently in CTCs and were not significantly higher in the progression cohort than in the baseline cohort (8% vs. 5%; *p* < 0.66).

Monitoring the *ESR1* mutations in the ctDNA can be a good tool to help guide therapy. The mutation of *ESR1* could improve PFS after a drug regimen with the ER down-modulator fulvestrant or by the addition of the mTOR inhibitor everolimus to exemestane [57,58]. Mutations in *ESR1* can be observed in ctDNA up to 6.7 months before progression in clinical settings, opening an extended window of opportunity for on-time therapeutic interventions [59]. In addition to ESR1 mutations, the following miRNAs have shown to be promising predictors for CDK4/6 inhibitor response or resistance in BC: miR-326, miR-29b-3p, miR-126, miR3613-3p, miR-432-5p, miR-223, and miR-106b [60]. Other biomarkers for CDK4/6 activity are the following ones: RB1, RB2 and RBL1, CDK4, CDK6, CCND1, CCND2, CCND3, CCNE1, CCNE2, CDKN2A, CDKN2B, CDKN1A, CDKN1B, FOXM1, AURKA, TP53, MDM2, and MDM4 [58]. Since liquid biopsy is the focus of this review, we referred to other reviews for details on those biomarkers [60,61].

As a result of these corroborating data, a ctDNA MRD assay has been developed, under the name NeXT Personal [51]. It is capable of detecting breast cancer relapses and has been associated with relapse-free survival. This assay efficiently detects high ctDNA rates during diagnosis and has a strong negative predictive value. Therefore, it has the potential to be used in de-escalation studies. Intervention prospective clinical trials aim to see if using NeXT could improve the accuracy of MRD detection [62]. The evolution of treatment decisions based on liquid biopsy findings started from the perspectives of early clinical trials. Some prospective clinical investigations have used cell-free tumor DNA for BC to predict therapy response. Only a few of them are cited here, since this is not the scope of this review: Umetani et al. in 2006 investigated the prognostic ability of cell-free DNA integrity (cfDI) to predict BC progression by examining at ALU260/111 ratios in 83 BC patients and 51 healthy controls [24]. The results showed that cfDI was significantly higher in stage II-IV patients vs. healthy controls [24]. Wang et al. studied the prognostic role of cfDI ALU260/111 and LINE1 266/97 to predict responses to neoadjuvant chemotherapy in a small group of BC patients. The results demonstrated that cfDI was significantly higher in BC patients after neoadjuvant chemotherapy (NACT) than before NACT. Higher cfDI correlated with tumor shrinkage and a reduction in Ki67 levels [63]. Cirmena et al. used cfDI from electrophoretic fragments as a biomarker of response to NACT. After NACT, cfDI was significantly higher in pathological complete responders compared to non-complete responders [64]. For a more comprehensive review of this topic, please refer to these reviews: [23,25].

With more advanced AI methods always becoming more available and gradually integrated in the medical field, liquid biopsy findings could evolve in terms of the paradigms of predictions in medical hospitals, through access to the confidential data of patients on hospital systems, from personal therapy information to the detection of biomarkers in blood, to produce in real-time solutions that are not yet available to us but that will become a reality in the near future.

## 2. Discussion and Conclusions

Liquid biopsy can be used in breast cancer from diagnosis through the treatment of metastasis (Table 1). Key components analyzed in liquid biopsy samples are CTCs, which separate from the initial cancer location and move into the blood circulation. Their presence could indicate metastatic activity and provide insights into tumor biology. cfDNA includes fragments of DNA released from cancer cells into the blood. Tumor-specific alterations in cfDNA, known as ctDNA, can be analyzed for mutation copy number variations.

Since solid tumors can evolve and mutate over time, which makes them resistant to targeted therapies the identification of a method that could detect this changes is a demanding priority.

Liquid biopsy could be used to promptly detect such alterations to predict therapy response. Emerging biomarkers in liquid biopsy, like exosome-derived RNA or circulating methylated DNA, should be further tested for their potential to predict cancer patients’ prognosis and therapy efficiency. Because these liquid biopsy biomarkers are not limited to specific mutations, they may better predict the heterogenous nature and evolution of breast cancer cells in the systemic circulation over time. AI methods have been developed in many fields, especially to aid breast cancer detection with MRI. Additionally, new AI methods have been predicted to constitute the future of predicting the efficacy of drugs such as ADCs [65]. Training AI with liquid-derived biopsy biomarkers for predicting responses to breast cancer therapies will be pivotal in the development of such diagnostic toolkits that could be implemented in prospective clinical trials tests, which would feed the model to achieve a good kit.

The implications of this review are multiple. First of all, for the early diagnosis of CTCs, cfDNA offers a non-invasive method for the early detection of BC. This could result in earlier interventions by medical personnel, which would result in improved outcomes for patients. CfDNA integrity and specific mutations (such as PI3KCA) have shown to be promising as early biomarkers. Using more sensitive methods like digital PCR could improve detection. Secondly, in post-treatment settings, monitoring cfDNA and ctDNA could improve the detection of MRD and predict recurrence ahead of time. This would give enough time for personalized adjustments and prompt intervention. The ctDNA was able to detect metastasis even before symptoms would appear, and methylation markers (e.g., RASSF1) in ctDNA were able to predict recurrence and response to treatment. Thirdly, as for the prediction of treatment response, LB can be versatile. In fact, it could help in the prediction of patients’ response to specific therapies, such as CDK4/6 inhibitors, endocrine therapy, and aromatase inhibitors. This allows doctors to have more tailored and effective treatment plans.

The advantage is that it is a less invasive method compared to traditional needle biopsy, capturing more accurately and comprehensively the heterogeneity of tumors and providing real-time monitoring of tumor evolution and response to therapy.

The challenges and limitations of liquid biopsy include (1) sensitivity and specificity, as current technologies may not detect very low levels of ctDNA or CTCs, and there is a risk of false positives/negatives; (2) standardization, as there is a need for common and reproducible methods/protocols for liquid biopsy analysis to ensure consistency and reliability across different labs; and (3) interpretation of results, as the clinical significance of some findings is still not completely understood, especially for early-stage cancer detection. There is ongoing medical research aiming to improve the specificity and sensitivity of liquid biopsies, develop new markers, and integrate these tests into routine clinical practice. As technologies advance, liquid biopsies may become a cornerstone of precision medical oncology, providing a less invasive and more overarching approach to the management of cancer. With the advancement in artificial intelligence (AI), it may soon be possible to integrate the data generated from cfDNA, ctDNA, and CTCs in the blood, urine, or even exhalations to create more accurate predictive methods to measure responses to therapy. This would require close collaboration between software engineers, scientists, and decision-maker physicians. The initial method should be tested with a small cohort of patients and then confirmed in gradually larger settings. Such AI-based prediction methods could be important tools to aid clinicians in making more informed therapy decisions. Finally, standardization efforts should be made to make sure that the same methods are used, including blood collection tubes, storage conditions, the type of qPCR, the length of long repetitive elements, and having the same assay conducted in the same manner across institutions to decrease biases. With the hope that, one day, healthcare would become accessible to everyone, we foresee these methods of liquid biopsy becoming more accessible and used in a larger population. The data generated from these diagnostic tools would also give rise to more ideas for strategies to best treat patients.

## Figures and Tables

**Table 1:** Liquid biopsy biomarkers for breast cancer.

Biomarker		Form	Setting	Model	Sample	Detection Method	Advantages	Limitations
**DNA**								
	cf DNA	DNA Fragments	Early detection	H	Plasma	RT-qPCR	High sensitivity and sensibility	Released from healthy and cancerous cells
	ct DNA	Methylation markers, copy number variation, rearrangements	All stages	H	Plasma	ddPCR, NGS, WGS, TARDIS	Identifies specific mutations, excellent sensitivity, residual disease in early and local advanced disease	Only known and limited number of mutations, low concentration in early disease
**ncRNAs (rRNA, tRNA, lncRNA, miRNA)**								
	miRNAs	20–25 Nucleotids	Diagnosis, prediction, prognosis	H	Plasma, Urine, Breast milk	NGS, RT-qPCR, microarray	Stable biomarker, in free or incapsulated form	Only few have miRNA been explored
**EV**								
	tdEVs	large vescicles	Advanced disease	H	Blood	Cell Search	More abundant than CTC, reflect tumor heterogenicity	Complex andtime-consuming techniques needed for their isolation,
	exoxomes			H	Plasma			
	microvescicles			H	Plasma			
**TEPs**								
		platelets	Diagnosis, prediction, prognosis	H	Blood	Low speed centrifugation	Useful RNA biosource	Sample collection, difficult isolation
**CTC**								
		CK/DAPI/EPCAM+ CD45−	Diagnosis, prediction, prognosis	H	Blood	Cell Search	Higher rate in MBC	Heterogenity, low number early setting

Abbreviations: cf DNA = circulant free DNA; ct DNA = circulant tumor DNA; RT-qPCR = Real Time Quantitative PCR; ddPCR = Digital Droplet PCR; NGS = Next Generation Sequencing; WGS = Whole Genome Sequencing; TARDIS = Targeted digital sequencing; ncRNAs = non coding RNAs; EV = extacellular vesicles; TEPs = tumor educated platelets; CTC = Circulant tumor Cells. H = Human model; CK = Citocheratin.

## Data Availability

No new data were created or analyzed in this study.

## Supplementary Materials

The following supporting information can be downloaded at https://www.mdpi.com/article/10.3390/genes16040443/s1, Figure S1: Diagram of literature research for liquid biopsy in breast cancer.

## Abbreviations

Breast Cancer (BC); Hormone Receptor-Positive (HR-positive); Hormone Receptor-Positive (HR+); Human Epidermal Growth Factor Receptor 2 (HER2); Triple-Negative Breast Cancer (TNBC); Endocrine Therapy (ET); Cyclin-Dependent Kinase 4/6 (CDK4/6) Inhibitors; Food and Drug Administration (FDA); Programmed Death-Ligand 1 (PD-L1); Circulating Tumor Cells (CTCs); Circulating Tumor DNA (ctDNA); Cell-Free DNA (cfDNA); Peripheral Blood Mononuclear Cells (PBMCs); Extracellular Vesicles (EVs); Loss of Heterozygosity (LOH); Single-Nucleotide Polymorphisms (SNPs); Polymerase Chain Reaction (PCR); Next-Generation Sequencing (NGS); Minimal Residual Disease (MRD); Aromatase Inhibitor (AI); Progression-Free Survival (PFS); Droplet Digital PCR (ddPCR); Tumor-Educated Platelets (TEPs); Long Noncoding RNAs (lncRNAs); MicroRNAs (miRNAs); Mechanistic Target of Rapamycin (mTOR); Antibody–Drug Conjugate (ADC); Cytosine–Phosphate–Guanine (CpG); Whole-Genome Sequencing (WGS); Targeted Digital Sequencing (TARDIS); and Reverse Transcription Quantitative PCR (RT-qPCR).
