# Supplementary figures and images for: Current Trends in Liquid Biopsy Tracking Resistance in Molecular Breast Cancer-Targeted Therapies
Source: Genes (Basel). 2025 Apr 9;16(4):443. doi: 10.3390/genes16040443 (PMC12027453; doi:10.3390/genes16040443)

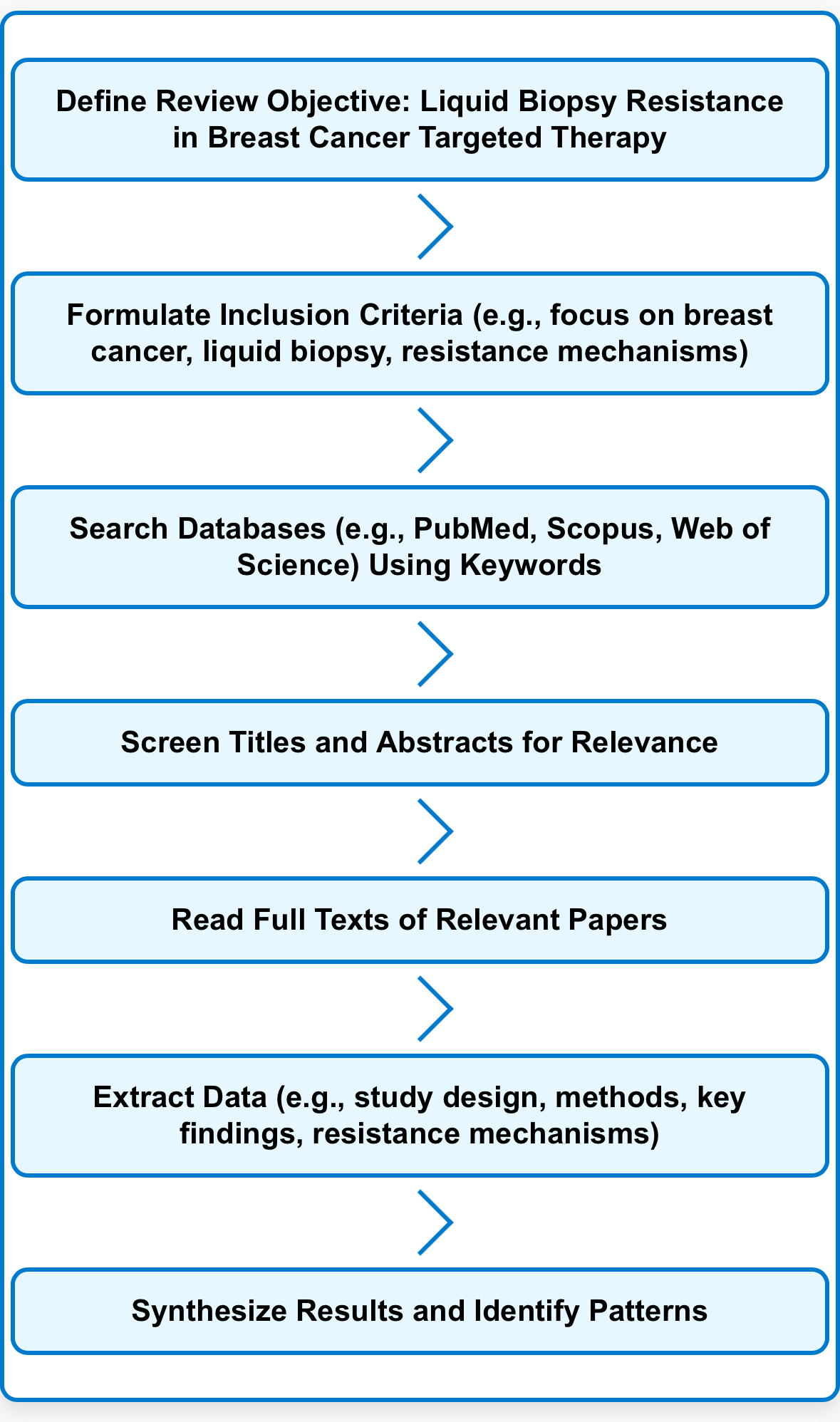


**Supplementary Figure S1**. Diagram of literature research for liquid biopsy in breast cancer.

Supplement: Supplementary file 1 [file genes-16-00443-s001.zip › genes-3508406-supplementary.docx]
